# Issues of methods and interpretation in the National Cancer Institute formaldehyde cohort study

**DOI:** 10.1186/1745-6673-9-22

**Published:** 2014-05-16

**Authors:** Gary M Marsh, Peter Morfeld, James J Collins, James Morel Symons

**Affiliations:** 1 Center for Occupational Biostatistics and Epidemiology and Department of Biostatistics, Graduate School of Public Health, University of Pittsburgh, 130 DeSoto Street, Pittsburgh, PA 15261, USA; 2 Institute and Policlinic for Occupational Medicine, Environmental Medicine and Preventive Research, University of Cologne, Cologne, Germany; 3 Institute for Occupational Epidemiology and Risk Assessment of Evonik Industries, Essen, Germany; 4 Epidemiology Department, 1803 Building, The Dow Chemical Company, Midland, MI, USA; 5 Epidemiology Program, Integrated Health Services, E.I. du Pont de Nemours and Company, Newark, DE, USA

**Keywords:** Formaldehyde, Nasopharyngeal cancer, Cohort mortality study, National Cancer Institute, Reanalyses, Silversmithing

## Abstract

In 2004, the International Agency for Research on Cancer (IARC) reclassified formaldehyde (FA) from a probable (Group 2A) to a known human carcinogen (Group 1) citing results for nasopharyngeal cancer (NPC) mortality from the follow-up through 1994 of the National Cancer Institute formaldehyde cohort study. To the contrary, in 2012, the Committee for Risk Assessment of the European Chemicals Agency disagreed with the proposal to classify FA as a known human carcinogen (Carc. 1A), proposing a lower but still protective category, namely as a substance which is presumed to have carcinogenic potential for humans (Carc. 1B). Thus, U.S. and European regulatory agencies currently disagree about the potential human carcinogenicity of FA.

In 2013, the National Cancer Institute reported results from their follow-up through 2004 of the formaldehyde cohort and concluded that the results continue to suggest a link between FA exposure and NPC. We discuss in this commentary why we believe that this interpretation is neither consistent with the available data from the most recent update of the National Cancer Institute cohort study nor with other research findings from that cohort, other large cohort studies and the series of publications by some of the current authors, including an independent study of one of the National Cancer Institute’s study plants.

Another serious concern relates to the incorrectness of the data from the follow-up through 1994 of the National Cancer Institute study stemming from incomplete mortality ascertainment. While these data were corrected by the National Cancer Institute in subsequent supplemental publications, incorrect data from the original publications have been cited extensively in recent causal evaluations of FA, including IARC. We conclude that the NCI publications that contain incorrect data from the incomplete 1994 mortality follow-up should be retracted entirely or corrected via published errata in the corresponding journals, and efforts should be made to re-analyze data from the 2004 follow-up of the NCI cohort study.

## Introduction

In 2004, the International Agency for Research on Cancer (IARC) reclassified formaldehyde (FA) from a probable (Group 2A) [1] to a known human carcinogen (Group 1) [2] citing the National Cancer Institute (NCI) cohort analyses for nasopharyngeal cancer mortality (NPC) [3]. Based on the same NCI findings, the Group 1 classification was upheld by IARC following the working group meeting for IARC Monograph Volume 100 F [4]. In Europe, the NCI FA cohort study as published by Hauptmann et al. [3] and evaluated by IARC 2006 [2] was also debated in detail to justify an appropriate cancer classification for FA according to the EU rules. The Committee for Risk Assessment of the European Chemicals Agency (RAC) disagreed with the proposal to classify FA as a known human carcinogen (Carc. 1A), proposing a lower but still protective category, namely as a substance which is presumed to have carcinogenic potential for humans (Carc. 1B). In 2011, in the US, the National Toxicology Program classified FA as a “known to be a human carcinogen” based in large part on the findings of the NCI study [5].

The French Agency for Food, Environmental and Occupational Health & Safety (ANSES)^a^, on behalf of the French Ministries of Health, the Environment and Labor, prepared a proposal for harmonized classification and labeling of FA^b^ after IARC’s decision to classify as a human carcinogen. The responsible Committee for Risk Assessment (RAC)^c^ of the European Chemicals Agency^d^ evaluated this proposal in 2012. The major discussion issue at RAC was the correct interpretation of the NCI FA cohort study on NPC mortality [3] taking into account all peer-reviewed publications that referred to this study and that explored the issue of NPC risk after FA exposure, in particular those publications that were published after 2004.

The Committee for Risk Assessment disagreed with the proposal to classify FA as known to have carcinogenic potential to humans (Carc. 1A), proposing a lower but still protective category, namely as a substance which is presumed to have carcinogenic potential for humans (Carc. 1B)^e^. Thus, U.S. and European regulatory agencies currently disagree about the potential human carcinogenicity of FA. Despite the fact that the classification systems of the different agencies are not identical the main message is clear: The RAC did not follow IARC's evaluation on human carcinogenicity.

### The National Cancer Institute formaldehyde cohort study

In June 2013, the NCI published the findings of its update through 2004 of mortality from solid tumors among workers in the US industry-wide FA study [6]. This study includes 10 plants and represents the largest cohort study of workers with potential exposure to FA [7]. The purpose of the Beane Freeman et al. update [6] was to extend the mortality follow-up through 2004 and to examine the associations among different exposure characterizations and mortality from several solid tumors. This study also included corrections to the earlier update of mortality through 1994 published in 2004 by Hauptmann et al. [8]. Beane Freeman et al. [6] claim that a persistent increased risk remains for NPC mortality associated with peak, average intensity and cumulative FA exposure metrics as reported in Hauptmann et al. [3], although this NPC risk was not reported by Blair et al. [7] in the original FA cohort analysis based on follow-up through 1979. The main conclusion from Beane Freeman et al. [6] is that the update through 2004 suggests a link between FA exposure and NPC mortality that is consistent with some case–control studies [9-14]. Aside from not statistically significantly elevated rate ratios for salivary gland cancer mortality, the authors observed no associations with mortality from other cancer types reported in other studies, including lung, laryngeal, nasal sinus and brain [1,4].

The NCI FA cohort study has a number of strengths including size, long follow-up time, several individual worker-level FA exposure metrics, partial adjustment for potential confounding factors, and the use of external and internal comparison populations. However, we have several concerns regarding the interpretation of the epidemiological findings from this study by Beane Freeman et al. [6], in particular, the purported link between FA exposure and NPC mortality. We believe that this interpretation is neither consistent with the available data nor with other research findings based on this group of US formaldehyde workers. The series of publications by the current authors and others present evidence [15-19]. Another serious concern relates to the incorrectness of the data reported by Hauptmann et al. [3,20] stemming from incomplete mortality follow-up that was first reported by Beane Freeman et al. [21]. While these data were corrected in subsequent supplemental publications [8,22], incorrect data from the original publications by Hauptmann et al. have been cited extensively in recent causal evaluations of FA, including IARC [4]. We note that IARC’s evaluation in 2004 [2] was mainly based on Hauptmann et al. [3,20].

This commentary describes our concerns and provides justification for continued re-analysis of the NCI cohort data on mortality. We will refer to three specific publications from updates of the NCI FA cohort: namely, the 1994 mortality update [3], corrections to the 1994 mortality update published as supplementary data [8,22] and the 2004 mortality update [6].

#### Issue 1 – Inconsistently reported cohort data

In 2010, Marsh et al. [15] described several major discrepancies between the number of observed deaths reported by Hauptmann et al. [3] and Beane Freeman et al. [22]. A main finding was that the corrected increase for total deaths by FA exposure status apparently equals 995, not 1006 as described by Beane Freeman [21]. We also found that when exposure is defined using a 2-year lag (used in the NCI analysis of lympho-hematopoietic cancers [3]), the percent increase in corrected numbers of deaths among FA “unexposed” subjects is approximately two times greater than that observed among FA “exposed” subjects for all deaths, all cancer deaths and all solid cancers [15]. Moreover, the basis of NCI’s revised count of 1006 deaths is neither evident nor reproducible from information provided. The most recent NCI publication by Beane Freeman et al. [6,8] did not clarify the discrepancy. Beane Freeman et al. [6] state (page 1016) that Hauptmann et al. [3] had missed 999 deaths and had counted four living subjects as deceased. They also reported 20 subjects whose age had exceeded the age of the oldest known decedent (102 years) as deceased. This information suggests that the corrected total death count should be 1015 (999-4 + 20), not the 1006 reported by Beane Freeman et al. [6]. Inconsistent with both numbers (1015 and 1006) that can be derived from Beane Freeman et al. [6], Table S1 in Beane Freeman et al. [8] yields a difference of 995 in comparison to Table two in Hauptmann et al. [3]. The identified differences are small and may have no substantial effect on estimates of relative risks, but the discrepancies between the original and corrected total numbers of deaths in the NCI cohort creates doubt whether all ascertainment errors were addressed by the NCI, as the consistent differential change in cause-specific numbers of deaths by exposure category cannot likely be explained as a chance occurrence. At the time of this publication, the NCI investigators have not presented an explicit clarification of the mortality ascertainment errors and the changes in total deaths and cause-specific deaths from the reported numbers presented in the Hauptmann et al. publications.

#### Issue 2 – Relevance of NCI’s corrected number of deaths

Tables 1, 2, 3 summarize key results from Hauptmann et al. [3], the only published corrections to this publication [8], and the updated mortality results reported by Beane Freeman et al. [6] for all causes of death combined and for NPC mortality in relation to FA exposure status based on external and internal comparisons. The inclusion of the claimed 1006 deaths missing in the analyses by Hauptmann et al. [3] had a major impact on the corrected risk estimates for total mortality, especially those based on internal cohort comparisons (Table 2). For example, *excesses* in total mortality ranging from 7% to 21% among workers in the two highest categories of ever-peak FA exposure (2 – 4 ppm and > 4 ppm) attenuated to *deficits* ranging from 4% to 15% in analyses that corrected the Hauptmann et al. publication [8] or updated the mortality follow-up [6], including some reduced risk estimates that were statistically significant. The overall standard mortality ratios (SMRs) were relatively unaffected by the corrections but corrected results for the 1994 mortality follow-up were more consistent with those reported in the most recent 2004 follow-up (Table 1). Thus, to clarify the record on the impact of these corrections, we submit that the incorrect NCI publications [3,20] should be retracted entirely or corrected via published errata in the corresponding journals.

**Table 1:** **Standardized mortality ratios and numbers of deaths for all causes combined by NCI update and FA exposure status**^**e**^

	**Formaldehyde exposure status**					
	**Non-exposed**			**Exposed**		
**NCI update**	**No. deaths**	**SMR**	**95% CI**	**No. deaths**	**SMR**	**95% CI**
All causes of death (ICD8: 001-999)						
NCI 1994 update^a.^	1,991	0.85*	0.81-0.89	6,495	0.96*	0.94-0.98
NCI 1994 update (corrected)^b.^	2,169	0.89*	0.86-0.93	7,312	1.02	0.99-1.04
NCI 2004 update^c.^	2,605	0.90*	0.87-0.94	11,346	1.03*	1.01-1.05
NPC deaths (ICD8: 147)^d.^						
NCI 1994 update^a.^	2	1.56	0.39-6.23	8	2.10	0.91-4.14
NCI 1994 update (corrected)^b.^	2	1.57	0.40-6.28	8	2.13	0.92-4.19
NCI 2004 update^c.^	2	1.45	0.17-5.25	9	1.84	0.84-3.49

^a.^Hauptmann et al. [3].

^b.^Beane-Freeman et al. [8].

^c.^Beane-Freeman et al. [6].

^d.^95% confidence intervals based on exact methods.

^e.^FA exposure status calculated using 15-year time lag.

*95% CI does not include 1.00.

**Table 2:** **Rate ratios and numbers of deaths for all causes combined by NCI update and peak FA exposure**^**f**^

**NCI update**	**Peak exposure (ppm)**								**p-value trend**^**d.**^	**p-value trend**^**e.**^
	**0**		**>0 - < 2.0**		**2.0 - < 4.0**		**≥ 4.0**			
	**RR 95% CI**	**No. deaths**	**RR 95% CI**	**No. deaths**	**RR 95% CI**	**No. deaths**	**RR 95% CI**	**No. deaths**		
NCI 1994 update^a.^	1.05	1,991	1.00 Baseline	2,554	1.21*	1,945	1.07*	1,996	0.013	0.014
NCI 1994 update (corrected)^b.^	1.02 0.85-1.04	2,169	1.00 Baseline	3,201	0.96 0.91-1.02	2,012	0.86* 0.81-0.91	2,099	<0.001	<0.001
NCI 2004 update^c.^	0.98 0.93-1.04	2,605	1.00 Baseline	4,996	0.95* 0.90-0.99	3,096	0.85* 0.81-0.89	3,254	<0.001	<0.001

^a.^Hauptmann et al. [20].

^b.^Beane-Freeman et al. [8].

^c.^Beane-Freeman et al. [6].

^d.^Two-sided likelihood ratio test (1df) of zero slope for continuous formaldehyde exposure among exposed person-years only.

^e.^Two-sided likelihood ratio test (1df) of zero slope for continuous formaldehyde exposure among unexposed and exposed person-years.

^f.^FA exposure metric calculated using 15-year time lag.

*95% CI does not include 1.00.

**Table 3:** **Rate ratios and numbers of deaths for NPC by NCI update and FA exposure metric**^**f**^

**NCI update**	**RR 95% CI**	**No. deaths**	**RR 95% CI**	**No. deaths**	**RR 95% CI**	**No. deaths**	**RR 95% CI**	**No. deaths**	**p-value trend**^**d.**^	**p-value trend**^**e.**^
Peak exposure (ppm)										
	0		> 0 - < 2.0		2.0 - < 4.0		4.0			
NCI 1994 update^a.^	1.00 Baseline	2	NA	0	NA	0	1.83	7	0.044	<0.001
NCI 1994 update (corrected)^b.^	1.00 Baseline	2	NA	0	NA	0	1.82 0.32-10.46	7	<0.001	0.05
NCI 2004 update^c.^	4.39 0.36-54.05	2	1.00 Baseline	1	NA	0	7.66 0.94-62.34	7	0.005	0.10
Average intensity of exposure (ppm)										
	0		0.1-0.4		0.5-0.9		≥ 1.0			
NCI 1994 update^a.^	1.00 Baseline	2	NA	0	1.38	1	1.67	6	0.126	0.066
NCI 1994 update (corrected)^b.^	1.00 Baseline	2	NA	0	0.37 0.03-4.63	1	1.66 0.29-9.48	6	0.07	0.14
NCI 2004 update^c.^	6.79 0.55-83.64	2	1.00 Baseline	1	2.44 0.15-39.07	1	11.54* 1.38-96.81	6	0.09	0.16
Cumulative exposure (ppm-years)										
	0		> 0 - < 1.5		1.5 - < 5.5		≥ 5.5			
NCI 1994 update^a.^	2.40	2	1.00 Baseline	3	1.19	1	4.14	3	0.029	0.025
NCI 1994 update (corrected)^b.^	2.41 0.35-16.70	2	1.00 Baseline	3	1.20 0.12-11.56	1	4.15 0.83-20.78	3	0.04	0.05
NCI 2004 update^c.^	1.87 0.30-11.67	2	1.00 Baseline	4	0.86 0.10-7.70	1	2.94 0.65-13.28	3	0.06	0.07

^a.^Hauptmann et al. [3].

^b.^Beane-Freeman et al. [8].

^c.^Beane-Freeman et al. [6].

^d.^Two-sided likelihood ratio test (1df) of zero slope for continuous formaldehyde exposure among exposed person-years only.

^e.^Two-sided likelihood ratio test (1df) of zero slope for continuous formaldehyde exposure among unexposed and exposed person-years.

^f.^All FA exposure metrics calculated using 15-year time lag.

*95% CI does not include 1.00.

#### Issue 3 – New NCI findings for NPC argue *against* an association with FA exposure

Beane Freeman et al. [6] reported results for updated mortality ascertainment through 2004 that provide evidence *against* an association between FA and NPC mortality. First, the most recent 10-year period of follow-up of the NCI FA cohort yielded only one additional NPC death. Further, this single NPC death occurred in the lowest exposure categories for the three FA exposure metrics evaluated by Beane Freeman et al. (Table 3). The observation of only one NPC death during a 10-year update of this large cohort is less than the corresponding number of expected NPC deaths for this period (1.14 as derived from information provided in the supplemental tables and the published report by Beane Freeman et al. [6,8]). First, this result by itself, argues against an association between FA exposure and NPC mortality. Second, unlike results reported in Hauptmann et al. [3] and the corrected analyses for the update through 1994 [8], the overall SMR for NPC among all FA-exposed workers in the most recent update through 2004 [6] is no longer greater than 2.00 and is not statistically significant (SMR = 1.84, 95% CI = 0.84-3.49) (Table 1). Third, the SMR for NPC among FA-exposed workers is now consistent with the corresponding SMR for all workers unexposed to FA (SMR = 1.45, 95% CI = 0.17-5.25), as the 95% confidence interval for FA-exposed workers is entirely contained within the confidence interval for FA-unexposed workers (Table 1).

Table 3 also shows that for the ever-peak exposure metric, the rate ratio (RR) for the two NPC deaths in the unexposed category compared with the single NPC death in the lowest exposed category is now 4.39 (95% CI = 0.36-54.05). This result is consistent with the RR for the highest category of ever-peak exposure (RR = 7.66, 95% CI = 0.94-62.34, 7 deaths), as the 95% confidence limits show a large overlap (heterogeneity chi-squared = 0.07 (d.f. = 1), p = 0.79). Likewise, RRs for the unexposed categories of average intensity and cumulative exposures (compared with the lowest exposure category for both metrics as the referent) were also elevated (RR = 6.79, 95% CI = 0.55-83.64 and RR = 1.87, 95% CI = 0.30-11.67, respectively). Finally, for average intensity and cumulative exposures, RRs for the moderate exposure categories (compared with the lowest exposure categories for both metrics) are now *less* than the RRs for the unexposed category (RR = 2.44, 95% CI = 0.15-39.07 and RR = 0.86, 95% CI = 0.10-7.70, respectively). These reported findings by Beane Freeman et al. [6] indicate that there is no meaningful difference in the RRs for NPC mortality between the unexposed and FA-exposed groups.

Compared with the original and corrected 1994 mortality updates [3,8], the 2004 mortality update reported by Beane Freeman et al. [6] provides little or no evidence of an exposure-response relationship for FA exposure and NPC mortality as shown in Table 3. First, the reported RRs are difficult to compare to RRs reported earlier due to the substantial methodological change of using the lowest exposure category as the reference rather than the use of the unexposed category as the reference in the previous 1994 update (discussed below under Issue 4). Second, for the same reason, the claim by Beane Freeman et al. [6] that a statistically significant trend (p = 0.005) for RRs for the ever-peak exposure analyses as based on FA-exposed workers only is difficult to compare to earlier findings (as discussed under Issue 5). Third, none of the trend tests incorporating all exposure categories in Beane Freeman et al. [6] are statistically significant. As noted by Beane Freeman et al. [6] (page 1018), *“RRs calculated using non-exposed as the referent were attenuated, which is also reflected in the tests for trend using all person-years…”.* This is evident in the results summarized in Table 3 that p-values for all trend tests estimated based on all unexposed and exposed person years are greater than 0.05. These results suggest that the mortality excesses for NPC among subjects *unexposed to FA* are simply due to chance or reflect the presence of some other exogenous factor outside of the study plants, consistent with findings reported by Marsh et al. [16] for Plant 1 (discussed below under Issue 7).

#### Issue 4 – Inappropriateness of excluding unexposed workers from the evaluation of exposure-response relationships

As noted throughout this commentary, Beane Freeman et al. [6] fails to address statistical evidence that non-exposure to FA is also associated with NPC mortality. The language used by Beane Freeman and colleagues in the published abstract states that previously observed excesses of NPC mortality “persisted” in the 2004 mortality update. This term implies that updated analyses were consistent with the previous reports and resulted in similar risk estimates for the three exposure metrics: peak, average intensity, and cumulative exposure as reported by Hauptmann et al. [3]. This interpretation is not accurate. In fact, the addition of one NPC death during the recent 10 years of additional follow-up changed the analytic approach for calculating RRs for both peak and average intensity exposures (Table 3). That is, the additional death was assigned to the "low" exposure group for each metric (0–2 ppm for ever-peak, and 0.1 - 0.4 ppm for average intensity exposure). In the previous mortality update through 1994 [3], and correction to this publication reported by Beane Freeman et al. [8], these "low" categories had zero observed NPC deaths, thus the "unexposed" category was used as the referent for internal RR analyses. Therefore, the estimates reported by Beane Freeman et al. [6] do not represent a "persistent" excess risk for ever-peak and average intensity exposures as they are relative to different referent categories for each exposure metric and must be interpreted in the context of the specified referent category.

It is inappropriate to exclude unexposed workers from internal analyses as done by Beane Freeman et al. [6]. All workers are from the same factories and, as noted by McLaughlin et al. [17] in a response to a letter by Hauptmann and Ronckers [23], lagging of FA exposure by 15 years results in contributions to the unexposed category from workers who were, in fact, exposed to FA. In fact, most of the person-time at risk allocated to the unexposed category represents years of follow-up of workers who were eventually exposed to FA. Thus, even speculated differences between unexposed and exposed workers with regard to unknown confounders cannot be used to justify the choice of the low-FA exposure category rather than the non-exposed category as the reference category for the calculation of relative risks [17].

#### Issue 5 - The trend tests used in the NCI 2004 updates produce misleading results and may be mis-specified

**Figure 1:**
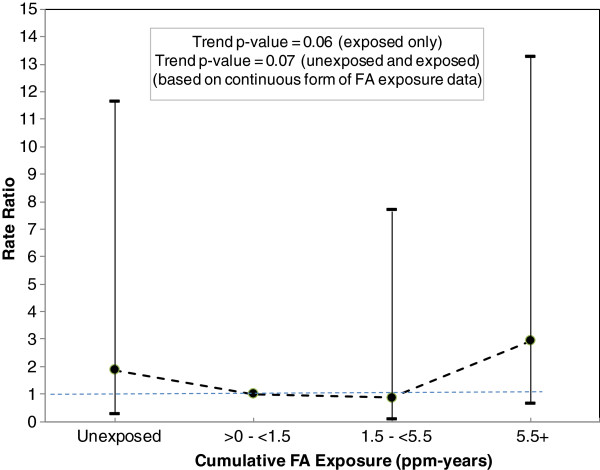
**Rate ratios and 95% confidence intervals for NPC by cumulative exposure (lagged 15 years) to FA (Beane Freeman et al. **[6]**).**

Beane Freeman et al. [6] used trend tests for categorical variables based on the likelihood ratio for the slope of the corresponding continuous variable, except for peak exposure, which was based only on categorical scores. Beane Freeman and colleagues provide no slope parameter estimates from the Poisson regression analysis of the continuous exposure values, and no indication that the authors evaluated the underlying assumptions and fit of the model. Assuming that the underlying assumption of linearity was met, Beane Freeman et al. could have used the slope parameter estimates to determine the increase in the RR per unit increase of FA exposure. In addition, Beane Freeman et al. placed the continuous variable-based trend test p-values in juxtaposition to the RRs derived from the categorical analyses presented, producing seemingly inconsistent results. For example, as shown in Table 3 the pattern of RRs across the four cumulative exposure categories are 1.87, 1.0 (baseline), 0.86 and 2.94. As shown in Figure 1, with or without the inclusion of the unexposed, these data provide no evidence on an increasing linear trend in RRs relative to increasing cumulative exposure to FA, yet trend test p-values are reported as 0.06 and 0.07 [6] without and with the unexposed category, respectively. Also, given that these p-values are 2-sided and a large number of person-years were accrued in the FA-unexposed category, it is also possible that these p-values indicate a nearly statistically significant inverse trend.

It is also quite possible that the inconsistent pattern of findings between the continuous and categorical data reflects the presence of an underlying non-linear relationship between log RR and continuous exposure. For example, if the underlying log RR vs. continuous exposure relationship was quadratic, the continuous model including the unexposed subjects could yield a marginally statistically significant positive slope in conjunction with no apparent trend or a U-shaped relationship in the RRs across the four exposure categories. An informative Poisson regression modeling approach would present an evaluation of the linearity assumption of the continuous model (i.e., linearity of log RR vs. continuous exposure), and if linearity is met, the corresponding slope estimates and confidence intervals.

In addition to the continuous model estimates for the continuous exposure variables, Beane Freeman et al. [6] should show trend tests based on the midpoints (or scores) of the categorical data presented in the tables. This categorization of the continuous variable is arbitrary, and the concurrent trend tests based on categorical midpoints (which also require underlying linearity with respect to log RR vs. midpoint score) directly reflect the reported and apparent pattern of categorical RRs presented in the tables. This avoids possible misrepresentation or confusion about trends (or lack thereof) based on the underlying and unseen continuous data. Non-linearity in the continuous log RR vs. continuous FA exposure relationship, such as a quadratic relationship, would invalidate the current trend tests. Another problem with the trend tests reported by Beane Freeman et al. [6] is the instability of the RR estimates based on only one observed death that occur in some baseline categories of the reported trends. This is especially problematic for the categorical analysis of NPC highest peak exposure (Table 3).

#### Issue 6 – Failure to recognize the important interaction structure between plant group and FA exposure

In regard to analyses reported by Marsh et al. [18], Beane Freeman et al. [6] state in the discussion section (page 1023), *“Based on two groups of plants (plant 1 vs. plants 2–10) and a continuous version of the originally categorical peak metric they demonstrated no significant interaction between plant group and peak exposure (p = 0.09) and adjustment for plant group did not markedly change the risk related to peak exposure*”. Beane Freeman et al. [6] further state, “*These results are consistent with our own analyses that showed no evidence of plant heterogeneity for a broad group of metrics, including peak exposure.”* First, the so-called “influence analysis” conducted by Beane Freeman and colleagues, amounts to excluding each of the 10 study plants one at a time to evaluate the consistency of the findings (a type of sensitivity analysis). This influence analysis was new to the 2004 mortality update [6] and presumably an attempt to address the heterogeneity in results for NPC by plant reported in the reanalyses of Marsh and Youk [19] and Marsh et al. [18]. Although Beane Freeman and colleagues address plant heterogeneity systematically, the authors neither correctly interpret the results of this analysis nor correctly interpret the results of the interaction evaluation performed by Marsh et al. [18].

Regarding the results of the reported influence analysis in the publication, Beane Freeman et al. [6] conclude that there is an apparent absence of plant heterogeneity upon removing Plant 1. However, the RR for NPC mortality in the highest category of ever-peak exposure decreased 56% from 7.66 for the 7 deaths including Plant 1, (95% CI = 0.94-62.34) to 3.36 for 2 deaths excluding Plant 1, (95% CI = 0.30-37.27). Moreover, the corresponding RR for the highest average intensity category of FA exposure decreased 65% from 11.54 for 6 deaths including Plant 1 (95% CI = 1.38-96.81) to 4.09 for 1 death excluding Plant 1, (95% CI = 0.25-66.0). These results for the influence analysis are quite similar to the RR for unexposed workers (RR = 4.39, 95% CI = 0.36-54.05). Further, when Plant 1 was included in the analysis and any other plant (from Plant 2 through Plant 10) was excluded, the results were similar to the results for analyses reported for all 10 plants. This pattern of findings in Beane Freeman et al. [6] is entirely consistent with the results of Marsh and Youk [19] which showed that the conclusion of a causal association was driven heavily by the large, statistically significant excess in NPC mortality risk for employees from Plant 1 for the highest ever-peak exposure category.

Regarding the interpretation by Beane Freeman et al. [6] of the results of the Marsh et al. [18] interaction evaluation, it is not true as stated by Beane Freeman and colleagues (page 1023) that the Marsh et al. analysis does not demonstrate a significant interaction between plant group and peak exposure. Rather, Marsh et al. [18] found that after dropping the main effect of plant group from the full interaction model (Model 9) the estimation process yielded more stable findings (Model 8) and the interaction between the plant group indicator and continuous peak exposure was found to be significant (p = 0.03). Unfortunately, a rigorous interaction analysis, such as that recommended and conducted by Marsh et al. [18] was not performed by Beane Freeman and colleagues.

#### Issue 7 – Misrepresented findings from the independent study of Plant 1

In 2007, Marsh et al. [16] reported findings from a 2003 update of their independent and expanded study of Plant 1 designed to investigate alternative explanations for the anomalous findings for NPC in this single plant. The results of the nested case–control study suggested that the large NPC mortality excess in Plant 1 may not be due to FA exposure, but rather reflects the influence of external employment in the ferrous and non-ferrous metal industries of the local area that entailed possible exposures to several suspected risk factors for upper respiratory system cancer (e.g., sulfuric acid mists, mineral acid, metal dusts and heat) [16].

In response to these findings, Beane Freeman [6] state (page 1022), *“Silversmithing was associated with risk of NPC, and Marsh et al.*[16]*concluded that the observed association between FA and NPC may actually be due to silversmithing. However, risks related to duration, cumulative and average intensity of exposure did not decrease when smoking and silversmithing or other metal work were added as adjustment variables, indicating that silversmithing does not confound the association between FA and NPC”.* We disagree with this interpretation because it assumes the presence of an association between FA and NPC that was not evident in the Marsh et al. Plant 1 study. In fact, Marsh et al. [16] observed considerable evidence that the overall NPC excess was not related to FA exposure in Plant 1. This additional evidence, summarized below, was not considered by Beane Freeman and colleagues in their discussion of the Marsh et al. Plant 1 study.

First, large, statistically significant NPC excesses were observed in both short-term (less than one year) and long-term workers (SMR = 5.35 and 4.59, respectively). Second, the seven NPC cases were associated with very short periods of Plant 1 employment (4 of 7 NPC cases worked less than one year, 5 of 7 cases worked less than five years), with low average intensity of FA exposure (the range of exposures for the 7 cases was 0.03-0.60 ppm with median of 0.14 ppm). Moreover, the NPC cases were concentrated among workers hired at Plant 1 during 1947–56, which was not the time period of highest FA exposure. Third, for average intensity of exposure, the metric most closely associated with the highest peak metric used in the NCI study, there was no evidence of an increasing NPC risk with increasing exposure (OR = 1.00, 11.41 and 2.18 for the categories <0.03 ppm (baseline), 0.03-0.159 ppm, and 0.16+ ppm, respectively). Finally, Beane Freeman et al. [6], observed mostly null findings for respiratory cancer sites other than NPC, in contrast to elevations in risk for these sites in the Marsh et al., Plant 1 study. For example, with the exceptions of cancer of the tongue and gum, local county rate-based SMRs in the Marsh et al. study were elevated for all upper respiratory cancer sites, including the nasal sinuses that do not seem to be penetrated by FA exposures [24].

#### Issue 8 – Failure of other major FA studies to find an increased risk of NPC

As noted by McLaughlin and Tarone in a commentary [25] and recent letter to the editor and response [26,27], Beane Freeman et al. [6] cite the NCI embalmer case–control study [28] in their discussion as evidence for a possible leukemia risk among FA-exposed workers, but ignore the absence of increased risk of NPC in the embalmer study. The odds ratio for NPC among embalmers was 0.10 (95% CI = 0.01-1.20), despite the fact that embalmers were reported to have the highest peak exposure to FA of any known occupation [28]. Also, the recent update of the NIOSH garment industry worker cohort [29] was not mentioned by Beane Freeman et al. [6] but only the older publication [30]. Interestingly, Meyers and colleagues still found no NPC deaths although follow-up of this FA exposed cohort was extended by 10 years to 2008 (number of expected deaths = 1.33). Finally, the most recent update of the British industry-wide FA study also found fewer NPC deaths than expected (1 death versus 2.0 expected) [31].

## Conclusions

The interpretation of an association between FA exposure and NPC mortality is neither consistent with the available data from the most recent mortality update of the FA cohort nor with other research findings from this cohort, other large cohort studies [29-31] and the series of publications by the current authors. The NCI publications [3,20] that contain incorrect data from the incomplete 1994 mortality follow-up should be retracted entirely or corrected via published errata in the corresponding journals. Efforts are underway by two of us (GM and PM) to re-analyze the cohort data from NCI’s 2004 follow-up with focus on a rigorous evaluation of the relationship between FA and NPC.

## Endnotes

^a^http://www.anses.fr

^b^Based on Regulation (EC) No 1272/2008 (CLP Regulation), Annex VI, Part 2

^c^http://echa.europa.eu/about-us/who-we-are/committee-for-risk-assessment

^d^ECHA, http://echa.europa.eu

^e^http://echa.europa.eu/web/guest/view-article/-/journal_content/c89bdb13-09e9-497c-8e73-ddae13a842c8)

## Abbreviations

ANSE: French agency for food, environmental and occupational health & safety; FA: Formaldehyde; IARC: International agency for research on cancer; NCI: National cancer institute; NIOSH: National institute for occupational safety and health; NPC: Nasopharyngeal cancer; RAC: Committee for risk assessment; RR: Relative risk or rate ratio; SMR: Standardized mortality ratio.

## Competing interests

The authors have no competing interest to declare.

## Authors’ contributions

GM and PM conceived of this commentary based on their experiences with past re-analyses of the NCI formaldehyde study and took lead roles in the drafting of the manuscript. JC and JMS contributed to the writing and editing of the draft manuscript. All authors read and approved the final manuscript.

## Authors’ information

GM is Professor of Biostatistics and Director of the Center for Occupational Biostatistics and Epidemiology at the University of Pittsburgh, Graduate School of Public Health. Since the 1980s, he has been involved in epidemiological research on the potential carcinogenicity of formaldehyde, including re-analyses of earlier updates of the NCI formaldehyde cohort and serving as principal investigator of an independent cohort study of workers from one of the NCI study plants.

PM is head of the Institute for Occupational Epidemiology and Risk Assessment of Evonik Industries AG. Evonik Industries and Cologne University have started a public-private partnership to conduct, and participate in investigations, research, and analyses relating to the health, safety, and epidemiological aspects of working conditions. The contract between Evonik Industries and Cologne University guarantees freedom of publication of all research work produced by the Evonik Institute. After his habilitation at Cologne University PM is teaching epidemiology and biostatistics at Cologne University. PM performed re-analyses of NCI’s industrial cohort formaldehyde study in cooperation with GM.

JMS is the Technical Supervisor of the Epidemiology Program for E.I. DuPont de Nemours and Company for which he conducts research on global occupational health and wellness issues.

JC is Director of the Epidemiology Program for the Dow Chemical Company for which he conducts research on global occupational health and wellness issues.

## References

[B1] IARC (International Agency for Research on Cancer) Wood dust and formaldehyde World Health Organization, ed. IARC monographs on the evaluation of the carcinogenic risk to humans. Vol. 62 1995 Lyon: IARC 35 215 http://monographs.iarc.fr/ENG/Monographs/vol62/mono62.pdf PMC7682304 7563584

[B2] IARC (International Agency for Research on Cancer) Formaldehyde, 2-butoxyethanol and 1-tert-butoxypropan-2-ol IARC monographs on the evaluation of carcinogenic risks to humans. Vol. 88 2006 Lyon: IARC 1 478 Available at: http://monographs.iarc.fr/ENG/Monographs/vol88/index PMC4781641 17366697

[B3] Hauptmann M Lubin JH Stewart PA Hayes RB Blair A Mortality from solid cancers among workers in formaldehyde industries Am J Epidemiol 2004 159 1117 1130 10.1093/aje/kwh174 15191929

[B4] IARC (International Agency for Research on Cancer) Formaldehyde World Health Organization, ed. IARC monographs on the evaluation of carcinogenic risks to humans. Vol. 100 F 2012 Lyon: IARC 401 435 Available at: http://monographs.iarc.fr/ENG/Monographs/vol100F/mono100F-29.pdf

[B5] U.S. National Toxicology Program. Report on Carcinogens 2011 Available from: http://ntp.niehs.nih.gov/?objectid=03C9AF75-E1BF-FF40-DBA9EC0928DF8B15

[B6] Beane Freeman LE Blair A Lubin JH Stewart PA Hayes RB Hoover RN Hauptmann M Mortality from solid tumors among workers in formaldehyde industries: an update of the NCI cohort Am J Ind Med 2013 56 1015 1026 10.1002/ajim.22214 23788167

[B7] Blair A Stewart P O'Berg M Gaffey W Walrath J Ward J Bales R Kaplan S Cubit D Mortality among industrial workers exposed to formaldehyde J Natl Cancer Inst 1986 76 1071 1084 3458945

[B8] Beane Freeman LE Blair A Lubin JH Stewart PA Hayes RB Hoover RN Hauptmann M Supplementary data Am J Ind Med 2013 56 1015 1026 10.1002/ajim.22214 23788167

[B9] Vaughan TL Strader C Davis S Daling JR Formaldehyde and cancers of the pharynx, sinus and nasal cavity: II Residential exposures Int J Cancer 1986 38 685 688 10.1002/ijc.2910380511 3770996

[B10] Vaughan TL Stewart PA Teschke K Lynch CF Swanson GM Lyon JL Berwick M Occupational exposure to formaldehyde and wood dust and nasopharyngeal carcinoma Occup Environ Med 2000 57 376 384 10.1136/oem.57.6.376 10810126 PMC1739963

[B11] Roush GC Walrath J Stayner LT Kaplan SA Flannery JT Blair A Nasopharyngeal cancer, sinonasal cancer, and occupations related to formaldehyde: a case–control study J Natl Cancer Inst 1987 79 1221 1224 3480373

[B12] Hayes RB Blair A Stewart PA Herrick RF Mahar H Mortality of U.S. embalmers and funeral directors Am J Ind Med 1990 18 641 652 10.1002/ajim.4700180603 2264563

[B13] West S Hildesheim A Dosemeci M Non-viral risk factors for nasopharyngeal carcinoma in the Philippines: results from a case–control study Int J Cancer 1993 55 722 727 10.1002/ijc.2910550504 7503957

[B14] Hildesheim A Dosemeci M Chan CC Chen CJ Cheng YJ Hsu MM Chen IH Mittl BF Sun B Levine PH Chen JY Brinton LA Yang CS Occupational exposure to wood, formaldehyde, and solvents and risk of nasopharyngeal carcinoma Cancer Epidemiol Biomarkers Prev 2001 10 1145 1153 11700262

[B15] Marsh GM Youk AO Morfeld P Collins JJ Symons JM Incomplete follow-up in the National Cancer Institute's formaldehyde worker study and the impact on subsequent reanalyses and causal evaluations Regul Toxicol Pharmacol 2010 58 233 236 10.1016/j.yrtph.2010.06.001 20553990

[B16] Marsh GM Youk AO Buchanich JM Erdal S Esmen NA Work in the metal industry and nasopharyngeal cancer mortality among formaldehyde-exposed workers Regul Toxicol Pharmacol 2007 48 308 319 10.1016/j.yrtph.2007.04.006 17544557

[B17] McLaughlin JK Lipworth L Tarone RE La Vecchia C Blot WJ Boffetta P Author reply to Hauptmann and Ronckers Int J Epidemiol 2010 39 1679 1680 10.1093/ije/dyp359

[B18] Marsh GM Youk AO Morfeld P Mis-specified and non-robust mortality risk models for nasopharyngeal cancer in the National Cancer Institute formaldehyde worker cohort study Regul Toxicol Pharmacol 2007 47 59 67 10.1016/j.yrtph.2006.07.007 17000042

[B19] Marsh GM Youk AO Reevaluation of mortality risks from nasopharyngeal cancer in the formaldehyde cohort study of the National Cancer Institute Regul Toxicol Pharmacol 2005 42 275 283 10.1016/j.yrtph.2005.05.003 15978711

[B20] Hauptmann M Lubin JH Stewart PA Hayes RB Blair A Mortality from lymphohematopoietic malignancies among workers in formaldehyde industries J Natl Cancer Inst 2003 95 1615 1623 10.1093/jnci/djg083 14600094

[B21] Beane Freeman LE Blair A Lubin JH Stewart PA Hayes RB Hoover RN Hauptmann M Mortality from lymphohematopoietic malignancies among workers in formaldehyde industries: the National Cancer Institute Cohort J Natl Cancer Inst 2009 101 751 761 10.1093/jnci/djp096 19436030 PMC2684555

[B22] Beane Freeman LE Blair A Lubin JH Stewart PA Hayes RB Hoover RN Hauptmann M Supplementary data J Natl Cancer Inst 2009 101 Available at: [http://jnci.oxfordjournals.org/content/suppl/2009/04/02/djp096.DC1/08 1103Rsupple.pdf] 10.1093/jnci/djp096 PMC2684555 19436030

[B23] Hauptmann M Ronckers CM RE: a further plea for adherence to the principles underlying science in general and the epidemiologic enterprise in particular Int J Epidemiol 2010 39 1677 1679 10.1093/ije/dyp361 20026593

[B24] Heck HA Casanova M Steinhagen W Everitt J Morgan K Popp J Formaldehyde toxicity: DNA-protein cross-linking studies in rats and nonhuman primates Nasal Carcinog Rodents: Relevance Human Risk 1989 40 159 164

[B25] McLaughlin JK Tarone RE False positives in cancer epidemiology Cancer Epidemiol Biomarkers Prev 2013 22 11 15 10.1158/1055-9965.EPI-12-0995 23118145

[B26] McLaughlin JK Tarone RE Mortality from solid tumors in the updated NCI formaldehyde cohort Am J Ind Med 2014 57 486 487 10.1002/ajim.22275 24501017

[B27] Beane Freeman LE Blair A Stewart PA Hayes RB Hoover RN Hauptmann M Response to Tarone and McLaughlin: RE: mortality from solid tumors in the updated NCI formaldehyde worker cohort Am J Ind Med 2014 57 4 488 489 10.1002/ajim.22294 24478120

[B28] Hauptmann M Stewart PA Lubin JH Beane Freeman LE Hornung RW Herrick RF Hoover RN Fraumeni JF Jr Blair A Hayes RB Mortality from lymphohematopoietic malignancies and brain cancer among embalmers exposed to formaldehyde J Natl Cancer Inst 2009 101 1696 1708 10.1093/jnci/djp416 19933446 PMC2794303

[B29] Meyers AR Pinkerton LE Hein MJ Cohort mortality study of garment industry workers exposed to formaldehyde: update and internal comparisons Am J Ind Med 2013 56 1027 1039 10.1002/ajim.22199 23788124

[B30] Pinkerton LE Hein MJ Stayner LT Mortality among a cohort of garment workers exposed to formaldehyde: an update Occup Environ Med 2004 61 193 200 10.1136/oem.2003.007476 14985513 PMC1740723

[B31] Coggon D Harris EC Poole J Palmer KT Extended follow-up of a cohort of British chemical workers exposed to formaldehyde J Natl Cancer Inst 2003 95 1608 1615 10.1093/jnci/djg046 14600093

